# Community-Effectiveness of Temephos for Dengue Vector Control: A Systematic Literature Review

**DOI:** 10.1371/journal.pntd.0004006

**Published:** 2015-09-15

**Authors:** Leyanna George, Audrey Lenhart, Joao Toledo, Adhara Lazaro, Wai Wai Han, Raman Velayudhan, Silvia Runge Ranzinger, Olaf Horstick

**Affiliations:** 1 Department of Community Medicine, Amrita Institute of Medical Sciences, Kochi, India; 2 US Centers for Disease Control and Prevention (CDC), Atlanta, Georgia, United States of America; 3 Ministry of Health, Brasilia, Brazil; 4 Consultant in Public Health, Manila, The Philippines; 5 Ministry of Health, Rangoon, Myanmar; 6 World Health Organization, Department for the Control of Neglected Tropical Diseases, Geneva, Switzerland; 7 Consultant to Special Programme for Research and Training in Tropical Diseases, World Health Organization, Geneva, Switzerland; 8 Consultant in Public Health, Ludwigsburg, Germany; 9 Institute of Public Health, University of Heidelberg, Heidelberg, Germany; Centers for Disease Control and Prevention, UNITED STATES

## Abstract

The application of the organophosphate larvicide temephos to water storage containers is one of the most commonly employed dengue vector control methods. This systematic literature review is to the knowledge of the authors the first that aims to assess the community-effectiveness of temephos in controlling both vectors and dengue transmission when delivered either as a single intervention or in combination with other interventions. A comprehensive literature search of 6 databases was performed (PubMed, WHOLIS, GIFT, CDSR, EMBASE, Wiley), grey literature and cross references were also screened for relevant studies. Data were extracted and methodological quality of the studies was assessed independently by two reviewers. 27 studies were included in this systematic review (11 single intervention studies and 16 combined intervention studies). All 11 single intervention studies showed consistently that using temephos led to a reduction in entomological indices. Although 11 of the 16 combined intervention studies showed that temephos application together with other chemical vector control methods also reduced entomological indices, this was either not sustained over time or–as in the five remaining studies—failed to reduce the immature stages. The community-effectiveness of temephos was found to be dependent on factors such as quality of delivery, water turnover rate, type of water, and environmental factors such as organic debris, temperature and exposure to sunlight. Timing of temephos deployment and its need for reapplication, along with behavioural factors such as the reluctance of its application to drinking water, and operational aspects such as cost, supplies, time and labour were further limitations identified in this review. In conclusion, when applied as a single intervention, temephos was found to be effective at suppressing entomological indices, however, the same effect has not been observed when temephos was applied in combination with other interventions. There is no evidence to suggest that temephos use is associated with reductions in dengue transmission.

## Author Summary

Dengue remains largely uncontrolled globally. Prevention and control relies on vector control methods, and good case management is key to reducing mortality. For vector control several methods exist, including biological and chemical interventions, and environmental modifications. The application of the organophosphate larvicide temephos to water storage containers is one of the most commonly employed dengue vector control methods. This systematic literature review assesses the community-effectiveness of temephos in controlling both vectors and dengue transmission when delivered either as a single intervention or in combination with other interventions. 27 studies were included in this systematic review (11 single intervention studies and 16 combined intervention studies). All 11 single intervention studies showed consistently that using temephos led to a reduction in entomological indices. Although 11 of the 16 combined intervention studies showed that temephos application together with other chemical vector control methods also reduced entomological indices, this was either not sustained over time or–as in the five remaining studies—failed to reduce the immature stages. Temephos was found to be effective at suppressing entomological indices, however, the same effect has not been observed when temephos was applied in combination with other interventions. There is no evidence to suggest that temephos use is associated with reductions in dengue transmission.

## Introduction

Vector control remains the only available intervention to prevent and control the transmission of dengue[1].Various vector control strategies aiming at controlling the principal vector of dengue, *Aedes aegypti*, are currently used with the intention of preventing the occurrence of dengue, or controlling outbreaks. These vector control measures often include the application of chemical or biological agents for the control of immature and adult mosquito stages, or environmental control methods that target mosquito breeding sites[2]. These vector control measures can be applied as single interventions or in combination.[3]. However, the efficacy and community-effectiveness of vector control strategies in terms of reductions in dengue transmission remain unclear, as previous systematic reviews have reported regarding the application of single intervention methods such as peridomestic space spraying and the use of *Bacillus thuringiensis israelensis*[4,5].

One of the most commonly employed methods for dengue vector controlis the use of the organophosphorous compound temephos (commercial name Abate) as a larvicide. Its use has been documented since 1965[6] in ponds, marshes and swamps at a dosage of 0.1–0.5 kg/ha for vector control in general, although fewer studies exist in relation to *Ae*. *aegypti*. Per the WHO Pesticides Evaluation Scheme, temephos can be used safely in potable water when the dosage does not exceed 56–112 g/ha (5.6–11,2 mg/m^2^) or 1 mg/l [7]. Moreover, the WHO hazard classification of temephos is “U”, meaning it is unlikely to cause acute hazard under conditions of normal usage[8].Temephos is a widely preferred tool for several reasons, including its ease and simplicity of application, selective killing of mosquito larvae and its long lasting effect when compared to traditional oil application methods[6].Temephos is commercially available in standardised preparations such as emulsifiable concentrates, dilute solutions, dusts and granules, including slow release formulations. It can be applied in different ways depending on the site and rate of application required. It can be delivered by hand or by injection through drip system devices or power sprayers. Temephos sand granules can be applied to household water storage containers of varying capacity by using a calibrated plastic spoon in order to administer a consistent dosage of 1ppm(1 ppm = 10^−6^ = 1 parts per million = 0,0001%) [9].

Temephos has widely been considered a cornerstone for controlling immature forms of *Ae*. *aegypti* yet while its efficacy has been demonstrated under laboratory conditions, comparable levels of efficacy are not necessarily replicated under field conditions[10].For the purpose of this article, efficacy under field conditions, including the use in ongoing control programmes, is referred to as community-effectiveness.

This systematic literature review assesses the community-effectiveness of temephos for controlling dengue vectors and dengue disease transmission when delivered in the field as a single intervention or in combination with other interventions.

## Methods

This systematic literature review follows the reporting guidelines described in the PRISMA statement[11].A comprehensive systematic literature search protocol was developed and agreed on by the authors consisting of six databases (PubMed, WHOLIS, GIFT, CDSR, EMBASE, Wiley).An additional review of grey literature,—screening the reference lists of the included publications as well as asking stakeholders about relevant literature—was performed, including theses, unpublished data, and other reports. The search was conducted until 15 June 2013. All sources fulfilling the predefined inclusion criteria and exclusion criteria were cross checked for additional references and these were included if again fulfilling the inclusion and exclusion criteria.

No language restrictions were applied and abstracts of publications in languages other than English were translated. The search included all studies irrespective of the year of publication.

The literature search strategy was based on four categories1) dengue vectors (*Aedes aegypti* or *Aedes albopictus)*,2) vector control intervention (temephos),3) dengue disease, and 4) dengue prevention and control. The search was conducted using the appropriate Medical Subject Heading (MeSH) terms followed by the Boolean operator “OR” for terms within each of the 4 categories, “AND” between categoriescombined with “free text” terms. The terms used for the 'vector' category included *Aedes aegypti* and *Aedes albopictus*. The search terms for the ‘vector control intervention’ category included insect control, vector control, mosquito control, larvicides, temephos, temefos and Abate. For the 'dengue disease' category, the terms dengue, dengue fever, DF, dengue hemorrhagic (haemorrhagic) fever, DHF, dengue shock syndrome and DSS were used. These terms were used in different combinations together with the terms prevention and control in order to broaden the search.

All titles and abstracts of potentially relevant articles were initially screened for the relevance of the research question and irrelevant articles as well as duplicates were excluded. The remaining articles that fulfilled the inclusion criteria were assessed, extracted and analysed using the full text of the study. The processes were conducted by two independent reviewers in consensus.

The inclusion criteria for this review were as follows: (i) Studies or programmes conducted with aim to prevent/control dengue; (ii) Studies with quantitative outcomes such as Breteau Index (BI), Container Index (CI), House Index (HI), larval mortality indicated by pupal skins, average number of positive containers per house, pupal index, indoor resting density, ovitrap index or dengue incidence; (iii) Community-effectiveness studies; iv) Peer reviewed studies with the study designs such as Randomised Control Trials (RCT), Cluster-Randomised Controlled Trials (CRCT), Non Randomised Control Trials (NRCT), Before and After studies, Studies with an Intervention and a Control area(Intervention studies); (v) Studies where temephos was used as a single intervention or in combination with other interventions.

Exclusion Criteria were: (i) Studies based in laboratory or semi-field settings; (ii) Studies where temephos was not used alone or as part of a combination intervention; (iii) Studies without clearly specified outcome measures; (iv) Cross-sectional studies, case series, reports, letters, newspaper articles, lectures, conference reports or abstracts.

After applying exclusion criteria, all included articles were categorised as either single or combination intervention studies and tabulated in an evidence table and stratified by type of study. Information on the study setting, objectives, design/sample size and study period as well as outcome measurements, main results and conclusions has been extracted.

Although considering the study quality by reflecting the study types as weighting tool for the discussion, no articles were excluded because of quality, taking the relative scarcity of relevant material into consideration.

To ensure the quality of this systematic review, the tool for assessment of systematic reviews (AMSTAR) was used[12].

## Results

**Fig 1:**
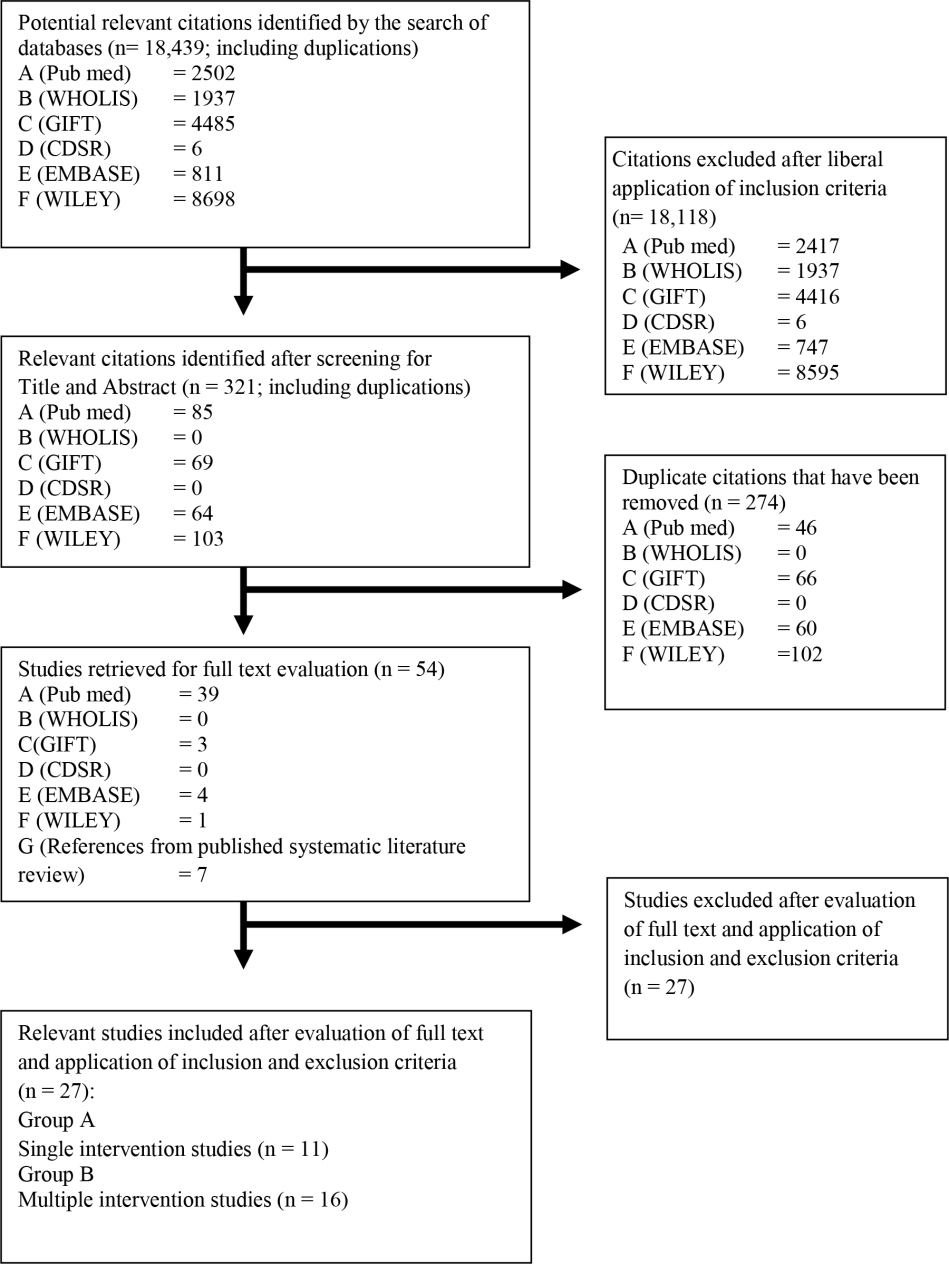
Flowchart of the systematic literature search.

The systematic literature search generated 18,439 potentially relevant citations. After screening by title and abstract, application of the inclusion criteria and removal of duplicates, 54 studies were retrieved for full text evaluation. After the final application of the inclusion and exclusion criteria, 20 articles were included and 7 further articles were added from the cross references and grey literature for a final total of 27included studies (Fig 1).

### General study characteristics

Of the27 studies, 14 were conducted in the Americas, 10 in South East Asia, one in Europe and two in the Western Pacific. Nine of the South East Asian studies were conducted in Thailand (Three by one researcher:Y.H.Bang).Even though no language restrictions were applied, all 27 articles retrieved were in English. All studies were publishedbetween1971 and 2012, of which seven studies were published between 2000 and 2012.

### Categorisation of selected studies

Of the 27 studies[13–39], 11 studies used temephos as a single intervention (Group A), while 15 studies used temephos in combination with other vector control methods (Group B). Only one study [21] tested temephos both alone and in combination, and this study has been included in the combination intervention group. The studies are described in detail according to the two groups(Tables 1 and 2).

**Table 1:** Summary of single intervention studies.

Study and (Ref No)	Reference	Setting	Objectives	Design, sample size and study Period	Intervention type	Outcome measurement	Main results	Conclusion of the study authors
[13]	Garelli FM *et al*.(2011)	Clorinda. Northern Argentina	Estimate the duration of residual effects of temephos Compare the effectiveness of temephos applied with spoons or in zip-lock bags Identify factors & processes that affect the decay of temephos residuality Describe the temporal pattern of *Ae*. *aegypti* immature infestations in containers treated with temephos	Before and after study 60 water tanks 2003 to 2008	1% temephos granular formulation was applied using i) spoons ii) inside perforated small zip-lock bags	Duration of residual effects of temephos according to water type & temephos application type Mean larval mortality	The median duration of residual effects of temephos applied using spoons was significantly lower than with zip-lock bags. Larval mortality was strongly affected by water type and type of temephos application. Water type and water turnover were highly significantly associated. Larval infestations reappeared nine weeks post-treatment and seven weeks after estimated loss of residuality.	Temephos residuality in the field was much shorter and variable than expected. Fast water turnover limits temephos residuality. Limited field residuality of temephos accounts for the inability of the larval control program to further reduce infestation levels with a treatment cycle period of 3 or 4 months.
[14]	Vezzani D *et al*.(2004)	Buenos Aires Cemetery, Argentina	To evaluate the efficacy of temephos in controlling *Ae*. *aegypti* in an environment with high urban container density.	Before and after study 400 to 1400 containers Nov to May 1998, 1999, 2000	1% temephos	Monthly % of breeding sites with *Ae*. *aegypti* Monthly % ofpositive ovitraps	Breeding sites decreased from 18.4% in the first study period (without temephos) to 2.2% in the second (two applications) and to 0.05% in the third (five applications). Ovitraps with eggs decreased from 17% in the first period to 5.8% in the second period, and to 2.9% in the third.	It is recommended to limit the use of temephos to prevent potential epidemics rather than for routine control.*Ae*. *aegypti* populations are highly susceptible to temephos.
[15]	Thavara U *et al*.(2004)	Kanchanaburi Province,Thailand	To evaluate the field efficacy of temephos zeolite formulation AZAI-SS in village trials against larval *Ae*. *aegypti* in water-storage containers	Before and after study 316 water storage containers 5 months	1% temephos zeolite granules.	Containers positive for larvae Acceptability of larvicide application by the residents	Containers with larvae which were treated had complete larval absence for 2 months. After 3, 4, and 5 months, 6–23% of the containers became positive despite the presence of temephos granules.	A single application of temephos zeolite granules at 1 ppm AI can provide highly satisfactory control of larval *Ae*. *aegypt*i in water-storage containers for at least 3 months in the field under normal water use practices.
[16]	Geevarghese G *et al*.(1977)	Poona, India	To study the field efficacy of Abate	Intervention control trial 127 houses intervention group and 105 houses control group. Dec 1973- Oct 1974	1% temephos	BI* CI HI	In one locality, *Ae*. *aegypti* population could be controlled for a period of 8 weeks in the dry season and for 12 weeks in the wet season by a single application. In another locality, the effect lasted only for 4 weeks in the dry season and for 6 weeks in the wet season.	Treatment of all potential breeding containers in an infested locality with 1 ppm Abate could control the species effectively, if applied at proper intervals.
[17]	Garza-Robledo AA *et al*.(2011)	Allende, Nuevo Leon, Mexico	To evaluate and compare the effectiveness of spinosad and temephos against mosquito larvae.	Intervention control study 12–17 tires Sep-Dec 2009	Spinosad effervescent Spinosad slow release Temephos sand granules	Number of *Ae*. *aegypti* and *albopictus* larvae	Spinosad and temephos were effective for up to 91 days post application.	Temephos is effective against *Aedes* larvae.
[18]	Bang YH *et al*.(1972)	Bangkok Thailand	To provide basic information on the i) effectiveness of larviciding ii) degree of control obtained, ii) personnel required iii) administration and management practicesappropriate to a large-scale control programme.	Intervention control studies 173 hectares1 year	Abate 1% sand granule formulation	BI Human bait for adult mosquito densities	The BI of 330 before temephos treatment was reduced to 3.9 after the first application. The BI rose from 1.9 to 67.8 during the 6 months following the fourth mass treatment. The time needed for routine larval inspections ranged from 3.7 to 7.1 min / house (average of 5.26 min). For the entire control period, the number of adult mosquitos caught in the treated area ranged from 0.2 to 2.1 (average, 1.04) /man hour.	Control of dengue vectors can be achieved by larviciding with 1% Abate sand-granules when applied just before the rainy season and repeated within 2 months.
[19]	Bang YH & Pant (1972)	Bangkok, Thailand	To compare the efficacy between two procedures (a) the mass treatment of all habitats followed by retreatment only after 1% of the total number of water jars have become positive (cyclic treatment) (b) mass treatment of all habitats followed by re-treatment of individual containers only when they become positive (positive source treatment)	Intervention control study 614 houses inhabited by 4100 persons 12 months	Abate sand granules applied as Cyclic treatment Mass treatment	Collection of adult mosquitoes landing on human bait Inspection of larval habitats. HI Egg collection by means of ovitraps	A high degree of control was achieved with reduction in adult density for an average of 3 months. There was 85% adult reduction using Abate, taking approximately 10 days to reach a very low level.	Reduction of both larval and adult density occurred with the combination of both methods. A combined method of cyclic mass treatment at 3 month intervals with simultaneous treatment of new habitats between the mass treatments would give better control.
[20]	Marina CF *et al*.(2011)	Southern Mexico	To determine the efficacy of spinosad and temephos on the inhibition of development of *Aedes* species	Intervention control trial 20 containers for each intervention 12 weeks each in dry and wet seasons	Oviposition traps were treated with:1 mg/L spinosad; 5 mg/L spinosad; 0.1 g temephos granules; 13 μL Bti suspension(VectoBac AS12);	Presence of larvae and pupae of *Aedes* spp. Total egg counts and percentage of eggs hatching from oviposition traps	*Ae*. *albopictus* was subordinate to *Ae*. *aegypti* during the dry season, but became dominant during the wet season. The complete absence of aquatic stages varied from 5 to 13 weeks for spinosad and 6 to 9 weeks for temephos depending on site, season and product concentration. Egg counts and % of eggs hatching of *Aedes* spp. increased significantly between the dry and wet seasons, but significant treatment differences were not detected.	Temephos granules and spinosad were both highly effective larvicides against *Ae*. *aegypti* and *Ae*. *albopictus*.
[21]	Morris CD *et al*. (1996)	Tire piles located in Land of Lakes, Florida	To study the control of *Ae*. *albopictus* in waste tire piles with varying concentrations of Abate	Intervention control study. Three piles of tires. 5 months	Granular formulations of temephos applied at either 0, 0.11, 0.56, 1.12. 11.21, or 22.42 kg AI/ha at a rate of 0.45 kg of dry corncob grit granules per 9.3m^2^	Larval mortality of *Ae*. *albopictus* Mean % reduction of *Ae*. *albopictus* larvae	Two days post treatment with temephos, larvae were reduced 90% at the 0.11 Kg AI/ha rate, 98% at the 0.56 Kg AI/ha rate and 100% at the 1.12, 11.21 and 22.42 kg AI/ha rates. Nearly 100% control was achieved for 8 weeks at the 0.5kg AI/ha rate, for 7 weeks at the 1.12 kg AI/ha rate in one replicate, and for at least 5 months at the 11.21 and 22.42 kg AI/ha rates.	Prolonged control of *Ae*. *albopictus* can be achieved at half the maximum allowable dosage. The ease of application, excellent distribution & penetration of the corncob grit granules suggest that both cost and manpower can be substantially reduced by the use of this formulation.
[22]	Nathan MB and Giglioli MEC(1982)	Cayman Brac and Little Cayman, West Indies	To describe the behaviour of *Ae*. *aegypti* on these islands and its successful eradication with Abate insecticide in 1970–1971.	Before and after study 49 premises (Little Cayman) & 575 premises (Cayman Brac)27 months	1.5% Abate 500E emulsion on indoor walls 1.5% Abate wettable powder suspension on outdoor walls and surfaces Abate 1% SG in water containers.	Premise index BI CI Ovitraps	While the first treatment was being conducted (May-June 1970) there was a considerable increase in foci with the arrival of wet weather. However, the initial treatment reduced the overall premise index from 31.5 to 2.6; although this index later rose again to 6.2%. Repeated surveys and retreatments eliminated all foci in 15 months.	In 1970–71 an intensive campaign against *Ae*. *aegypti* adults and larvae resulted in eradication of this mosquito from the Lesser Cayman Islands.
[23]	Chadee DD(2009)	Trinidad, West Indies	To study the effect of pre-seasonal focal treatment on the population density of *Ae*. *aegypti* during the first 2 months of the rainy season	Intervention control study 9403 containers 25 weeks	1% temephos	BI HI CI PI Ovitraps	Following focal treatment during the month of April, the *Ae*. *aegypti* population declined significantly from a BI of 19 to 6 & PI from 1.23 to 0.35 in May, while in control sites the BI increased from 23 to 38 & PI from 0.96 to 2.00 in August. The *Ae*.*aegypti* population did not return to pre-treatment levels until 9–11 weeks post treatment.	The timely application of pre-seasonal focal treatment with temephos together with standard control measures, such as source reduction, can reduce the BI to <5 and the PI to <0.71. It also extended the duration of vector suppression.

**Table 2:** Summary of combined intervention studies.

Study	Reference	Setting	Objectives	Design, sample size, and study Period	Intervention type	Outcome measurement	Main results	Conclusion of the study authors
24	Shriram AN *et al*.(2009)	Brookshabad,Andaman and Nicobar Islands, India	To assess the *Aedes* spp. infestation and subsequently test the efficacy of a community-based approach to control *Ae*. *Aegypti*	Before and after study 533 containers from 104 premises Aug 2006—Oct 2007	Community centred approach-IEC Environmental management-source reduction Anti-larval campaign using temephos 50% EC	BI CI HI	Post-temephos application, the number of containers supporting *Ae*. *aegypti* breeding reduced significantly within 1 month. The BI and HI dropped from 104.8 to 2.7 and 44.2 to 2.6, respectively. Thereafter, the indices remained at zero until the completion of the study.	Temephos applied through a community- centred approach is an effective control measure for these islands. Close monitoring of this approach is warranted for long term sustainability.
25	Chaikoolvatana A *et al*.(2008)	Si Sa Ket, North Eastern Thailand	i) To compare the effectiveness of currentlyavailable interventions for dengue vector control ii) To measure the basic knowledge of the local population about the use of different interventions for dengue vector control	Intervention control study 568 households 7 months (March-Sept. 2007)	Pyrethroid ULV space thermal fogging 1% temephos sand granules iii) Combination of both interventions	BI CI HI DHF cases Cost of different interventions Survey of knowledge of the people and their sources of information	1% w/w temephos sand granules alone was the most effective intervention in terms of BI reduction and cost. Basic knowledge of dengue was positively associated with reductions in BI.	This study illustrated the importance of the role of public health workers in dengue vector control and highlighted the need to engage communities in health issues at the local level.
26	Laws ER Jr *et al*.(1968)	Manati, Puerto Rico	To conduct medical and entomological surveillance during the introduction of Abate in the field.	Before and after study 401 premises19 months	1% Abate SG 2.5% malathion spraying used in non potable water & surrounding areas	Reasons for the refusal of Abate usage % of premises positive for *Ae*. *aegypti* larvae Abate concentrations in water and human urine	Use of Abate and malathion spraying gradually achieved control of *Ae*. *aegypti* during the first 4 months of treatment and control was maintained thereafter. With minor exceptions, residents readily accepted this use of Abate.	Abate is considered safe for full-scale field use when applied according to recommended dosages.
27	Gürtler RE *et al*.(2009)	Clorinda, northeastern Argentina	To describe the implemented intervention & assess the long-term effects on larval indices and reported incidence of dengue during the five-yearperiod.	Before and after study 448 households 5 years (2003–07)	1% temephos sand granules applied in non-potable water Occasional Bti (VectobacR, Bayer) treatment of water containers iii) Minimal source reduction Emergency response indoor and outdoor ULV spraying Health education	HI BI	BI declined significantly in nearly all focal cycles compared to pre-intervention levels. Temephos residuality was shorter than expected, with infestations in treated containers detected within 6 weeks of treatment.	Control interventions exerted significant impacts on larval indices but failed to keep them below target levels every summer. For further improvement, a shift is needed towards a multifaceted program with intensified coverage and long-term sustainability.
28	Lardeux F *et al*.(2002)	Rangiroa Atoll, French Polynesia	To describe mosquito larval habitats, adult mosquito levels, and community awareness, acceptability and participation in the mosquito control intervention	Intervention control study 150–250 premises 18 months	Sealing cisterns/wells with mosquito screening1% temephos application in potable water Application of polystyrene beads into cisterns/wells iv) introducing fish into non potable water.IEC	Mean number of biting *Aedes*/ human /15 min Larval indices Community acceptance survey	Entomological indices from human bait collections & larval surveys indicated that mosquito populations were reduced significantly, compared with control villages. The mosquito control remained effective for 6 months after treatment. Effects of the treatment were noticed by the inhabitants in terms of a reduction in the number of mosquito bites.	Control programs may succeed in the long-term only if strong political decisions are taken at the village level in order to maintain sustainability.
29	Rizzo N *et al*.(2012)	Guatemala	To test the efficacy, cost and feasibility of a combined approach of insecticide treated materials alone and in combination with targeted breeding site interventions.	Cluster RCT20 clusters (1835 houses)18 months	PermaNet 2.0 (deltamethrin at 55 mg/m2) curtains and jar covers iCombination of PermaNet curtains and jar covers, 1% temephos and source reduction	Total production of *Aedes* pupae, Pupae per person index (PPI)HIBICI	The curtains and jar covers intervention did not produce a detectable impact on entomological indices. When it was combined with the targeted application of temephos, the interventions led to significant differences in the total number of pupae and HI between intervention and control clusters and borderline differences in the PPI and BI.	The combination of insecticide treated curtains and jar covers together with interventions targeting productive container types can significantly reduce entomological indices.
30	Favier C *et al*.(2006)	Brasilia, Brazil	To determine the influence of climate and environmental vector control with or without insecticide on *Ae*. *aegypti* larval indices and pupal density	Intervention control study 1015 premises Dec 1997- May 1999	Control zone with environmental management (EM) alone Methoprene-S (Altosid) and EMBti (Mosquito dunks) and EMTemephos (Abate) and EM	% premises with potential breeding sites BI HI CI Pupal density per premise and positive container	Environmental vector control strategies dramatically decreased infestation in the study areas. No significant differences could be detected between control strategies with or without insecticide.	In a moderately infested area, insecticides do not improve upon environmental vector control. Infestations could be further reduced by focusing on residences an containers particularly at risk.
31	Wheeler AS *et al*.(2009)	Grand Cayman Island	To describe the control efforts used to contain the *Aedes albopictus* infestation on Grand Cayman up until the end of 2001.	Before and after study 5 key areas 1997–2001	Focal treatment around infestations with:Permethrin space spray(Permanone RTU, 4%) Residual application of lambda–cyhalothrin (Demand CS, 10%) on external walls and eaves and nearby vegetation Truck-mounted ULV with fenthion Granular larvicide treatments to all outdoor containers alternating (on a 2-week rotation) temephos (Abate) or methoprene (Altosid)	BI Oviposition indices	For a 2-year period after the initial introduction, the abundance of this species remained low (BI ranging from 0.6 to 5). However, in 1999, *Ae*. *albopictus* became firmly established (BI of 5 to 5.5). By the end of 2001, it was apparent that the control methods utilized had failed to stop the spread of this species.	This work describes the control methods utilized to control *Ae*. *albopictus* and discusses possible reasons for the failure of the elimination campaign, mainly related tolack of resources and manpower.
32	Bang YH *et al*.(1972)	Bang Sue,Bangkok, Thailand	To determine the efficacy of malathion thermal fogs, applied soon after larviciding with 10% Abate SG, on the suppression of *Ae*. *aegypti*.	Intervention control study Site A: 172 houses Site B: 132 houses Control: 150 houses 27 weeks	Site A: 4% malathion thermal fogs and1% Abate SGSite B: 1% Abate SG iii) Control: no treatment	Density of adult *Ae*. *aegypti* and number of habitats positive for larvae Ovitraps Adult mosquitoes landing on human bait	In all areas treated with Abate, irrespective of additional malathion fogging, the adult mosquito population did not begin to increase until 6–7 weeks post-treatment. Larval breeding control lasted approximately 8 weeks.	A single application of the larvicide alone might be sufficient to control mosquito populations. However, treatments with Abate larvicide or Abate in conjunction with insecticide fogs have yet to be studied in areas of an actual epidemic.
33	Donalisio MRC *et al*.(2002)	Americana São Paulo, Brazil	To evaluate the efficacy of temephos for the control of the *Ae*.*aegypti* larvae	Randomized control trial Experimental area: 17,994 houses(665 blocks); Control area: 37,955 houses (1775 blocks)10 months	Experimental area: 1% temephos,source reduction, IEC Control area: source reduction, IEC	BI CI	Areas that received temephos application presented similar levels of *Ae*. *aegypti* larval infestation as the untreated area. The larval infestation varied with the degree of rainfall but not with temperature.	A false sense of complacency created by the larvicidal treatment may have contributed to increased negligence in the physical elimination of breeding sites by communities. The effectiveness of temephos was transient (lasting around 3 weeks) and could have been influenced by the overflowing of water and consequent dilution factors in non-removable containers.
34	Wang CH *et al*. (2000)	Small Liu-Chiu Isle, Taiwan	To report the strategy and the effectiveness of an integrated vector control program that aimed to prevent recurrences of dengue and DHF epidemics	Before and after study 8 villages 1989 to 1996	Larvivorus fish *Bti* Temephos Source reduction Improvement of water storage containers Health education	BI CI	Before implementation in 1988, 24% of water containers were infested with *Ae*. *aegypti*. In 1989, the BI for the entire island was 53.9, as compared to 1.2 in 1996. ln 4 villages located at the southwest and middle of the island, *Ae*. *aegypti* was nearly eliminated. Before the implementation of program, *Ae*. *aegypti* was dominant in containers both inside and outside the household, but after the implementation, *Ae*. *albopictus* became dominant outside.	Motivating community members to participate in integrated control programs was more effective for long-term control of dengue and dengue vectors.
35	Gould DJ *et al*.(1971)	Koh Samui, Thailand	To assess vector densities, incidence of dengue virus infections in vector populations	Before and after study 5 villages 10 months	Abate 4-E Ground applied malathion fogs	CI HI Adult mosquitoes collected per man hour	An island-wide post-intervention reduction in both the larval and adult populations of *Ae*. *aegypti* was observed. The duration of pronounced *Ae*. *aegypti* suppression varied from 2 to 8 weeks. The Abate treatments were also effective in controlling the breeding of *Ae*. *albopictus* in artificial containers inside and around houses and *Ae*. *albopictus* adults were temporarily suppressed in those areas where malathion fogs were applied.	There was no indication that the temporary reduction in the *Ae*. *aegypti* population resulted in any significant invasion of domestic habitats of that species by *Ae*. *albopictus*.
36	Phanthumachinda B(1985)	Phanus Nikhom district, Chonburi province, Thailand	To develop mechanisms for individual and community participation in the prevention and control of DHF through source reduction.	Before and after study Site 1:2921 premises Site 2 :675 premises Site 3 :549 premises 1983–84	Group educational activities 1% temephos SG (replaced later by methoprene) Source reduction	Larval surveys BI	Initial BI reduction after the 1st mass campaign was 45–60%, reaching a maximum of 70% for site 1 (BI 133, baseline 525), 78% for site 2 (BI 115, baseline 525) and site 3 63% (BI 165, baseline 452).BI reduction after the 2nd mass campaign was not as significant as compared to the initial one due to the onset of the rainy season. In each study area, the rainy season rise in BI was temporarily stopped in September by the 3rd mass campaign in August.	Although BI was reduced, infestation levels still remained high. Periodical larviciding with temephos or methoprene provided by the government were found to be neither economical nor practicable.
37	Eamchan P *et al*.(1989)	Nakhon Ratchasima, Thailand	To describe epidemiology and control of dengue virus infections in rural villages in northeast Thailand.	Before and after study 10 villages 2 months	Malathion fogging 1% Abate sand granules IEC Covering water jars	HI BI CI Adult *Aedes* landing rate	The HI dropped from 67 to 20, the CI decreased from 30 to 5 and the BI decreased from 221 to 33.	Efforts at controlling mosquitoes using malathion, 1% Abate SG and educating the public was met with only limited success.
38	Swaddiwudhipong W *et al*.(1992)	Mae Sot District, Tak province, Northern Thailand	To study the effect of health education on a community based DHF vector control program	Before and after study 6,341 houses with 20,283 inhabitants 1989–90	IEC Temephos 1% sand granules ULV spraying Public piped water system Larvivorous fish	HI CI BI	During the study period, water storage containers accounted for about 90% of the total breeding places. Vector density was reduced following the household intervention, but it recovered by 3 months later. The household visit by the health team trained to educate people in the community had only a moderate effect on vector control.	Vector control strategies should comprise not only eliminating the existing breeding sites but also preventing the creation of new ones.
39	Vanlerberghe V *et al*. (2009)	Guantanamo,Cuba	To assess the effectiveness of an integrated community based environmental management strategy to control *Ae*. *aegypti*, compared with a routine strategy.	Cluster RCT 16 intervention clusters (400 houses total) and 16 control clusters (400 houses total) 1 year	Control clusters (routine control programme):Source reduction Selective adulticiding Temephos Health education Intervention clusters: routine *Aedes* control programme combined with community based environmental management.	HI BI Pupae per person	At baseline, the *Aedes* infestation levels were comparable between intervention and control clusters. At the end of the intervention, these indiceswere significantly lower in the intervention clusters: rate ratio for house indices 0.49 (95% confidence interval 0.27 to 0.88) and rate ratio for pupae per inhabitant 0.27 (0.09 to 0.76).	A community based environmental management strategy embedded in a routine control programme was effective at reducing levels of *Aedes* infestation.

*BI = Breteau Index; CI = Container Index; HI = House Index

### Studies using temephos as a single intervention (Group A)

#### Study designs

Of the 11 studies, seven were Intervention Control studies[16,17,18,19,20,21,23] and four had Before-and-After studies design [13,14,15,22] (Table 1 for more detailed description).

#### Outcome measures

In most studies, the classical immature entomological indices such as the House Index [16,22,23], Container Index[13,15,16,18,19,22,23]and Breteau Index [16,19,22,23] were used to measure the outcomes. Pupae/Person Index [13,23] and adult mosquito density [16,18,19] were also measured. Reduction of breeding sites [14] and positive ovitraps were also measured [14,19,20,22,23]. Mean number of (total, late and early stage) *Aedes* larvae[13,17,19,20], % prevalence of *Aedes*[20]and mean % reduction of *Ae*.*albopictus*[21] were some of the other outcome measures that were analysed. Other findings such as quantity of temephos, labour and time required for its application were analysed only in study number[19].

Factors affecting the residual efficacy of temephos (such as temperature, organic debris, exposure to sunlight, water use patterns, maintenance of water storage containers) and level of acceptance or resistance to use of temephos among household residents were analysed only in one study [15], and expenditure for temephos application was calculated only in study number[19]. Study number [13] was the only study that analysed the association between larval mortality and factors such as the mode of application of temephos using spoons or zip-lock bags, different water types, effects of sunlight and type of containers used for water storage.

#### Interventions

The 11 studies had different temephos application periodicity, with four studies [14,16,20,23] conducted according to the dry and wet seasons while study number[23]applied temephos as a pre-seasonal focal treatment. Of the 11 included studies, five had used temephos in the form of sand granules [13,17,18,19,22], while study number [15] experimented with temephos zeolite formulations. Four of the studies [14,16, 20,23] did not mention the type of formulation and only mentioned the dose (as parts per million (10−^6^ or ppm), and in study number [21] different dosages of temephos were compared. In most of these studies, temephos was applied at a dose of 1 ppm based on the size of the containers [18,19]. However, in study number [23], temephos was applied based on the actual volume of water present at the time of inspection rather than the total holding volume of the containers. The mode of temephos application differed across studies and included such diverse methods as the use of permeable zip-lock bags [13], plastic spoons [19], granules placed in a well-perforated plastic micro-centrifuge tube that allowed the granules to be removed during monitoring procedures [20], corncob grit granules [21] and the application of wettable powder to walls[22]. In studies where temephos was compared to other insecticides [17,20], it was often compared to Spinosad [17,20] and *Bti*[20].

Sources of water and the container material also differed across studies. Study number [13] compared the effects of temephos in rain water, piped water or both and also its effects in fibrocement or plastic containers. Studies number [18,19] applied temephos in water which was not used for the purpose of drinking.

#### Community-effectiveness

All 11 studies reported a post-intervention reduction in the immature stages when compared to their respective control group. It was observed that the treated sources were free of larvae for a variable period of time depending on the season of application [16,20,23], number of applications [14,19], dosage of temephos[21], procedure of control [19], and method of application with respect to the source of water and container for storage of water [13].

For example, it was observed in study number [16] that the *Aedes* population was effectively controlled for 4–8 weeks in the dry season with no larvae and 6–12 weeks in wet season. In study number [20], there was an absolute inhibition of mosquito development for 6–9 weeks in the dry season and 9 weeks in the wet season. However, study [24] showed a pre-seasonal focal treatment before the rains reduced indices for 9–11 weeks, after which they returned to pre-treatment levels.

When considering the number of applications, study[14] reported that as the number of applications increased to five, it led to the elimination of the immature population. Study[19]found that a total of four applications could be effective at suppressing entomological indices, but concluded that two treatments would most likely be effective if applied just before the rainy season and repeated again within 2 months.

Variable effectiveness of temephos according to dosage was reported in study number [21], when it was applied at half the maximum allowable dose (11.21kg AI/ha)it provided100% protection for 5 months, with excellent control at rates as low as 0.56kg AI/ha(100% reduction sustained for 8 weeks). Study number[17] reported 100% effectiveness for 90 days when temephos was applied at the dose of 20g/200 litres of water.

Study number [19] observed that cyclic mass treatment with temephos required a greater amount of larvicide, while the targeting of treatment to positive containers required a greater number of man-hours. The effective control period was 2.5 to 5 months with an average of 3 months. The study concluded that a combined method of cyclic mass treatment at 3 month intervals with simultaneous treatment of new habitats between the mass treatments would offer the best control.

Factors such as temperature, organic debris, and ultraviolet light from sunlight that possibly degraded the active compound were investigated in study numbered [15]. These factors coupled with rapid water use, draining, and refilling of containers shortened the residual effectiveness of temephos [15]. This study also pointed out that other factors such as regular supply and budget allocation for the purchase of temephos are major factors affecting the success of a programme. Likewise, in study number[13], the median duration of the temephos effect was two to three weeks, with the duration of the residuality affected by water management practices (water type and water turnover), which were found to be significantly associated with reduced larval mortality. This same study found that temephos applied inside zip-lock bags provided longer residual activity as compared to spoon based applications[13].

Study number[22] illustrated the community-effectiveness of temephos by eliminating *Ae*. *aegypti* from an island by applying temephos intensely as both a residual insecticide on walls of houses as well as the treatment of breeding sites. Through this combination of temephos treatments, *Ae*. *aegypti* was eliminated within 15 months of the implementation of the campaign.

#### Acceptability

Very few studies evaluated the acceptability of temephos applications[13,15]. As per study [13], acceptability of householders for temephos application via zip-lock bags was higher than spoon based application since it was easy to apply and inexpensive to implement. In study [15], 89% of the participants refused to use temephos sand granules due to the unpleasant odour and increase in water turbidity and perceived safety risk. No studies analysed the operational challenges associated with temephos application or the undesirable consequences caused by it.

### Studies using temephos in combination with other interventions (Group B)

#### Study designs

Of 16 studies in this group, nine were Before-and-After studies [24,26,27,31, 34,35,36,37,38], four Intervention Control Studies [25,28,30,32] and three Cluster Randomised Trials [29,33,39].All 16 studies used households as the sampling unit for the application of temephos in various domestic breeding sites. Two studies were conducted on islands [31,35], seven in cities or towns[26, 27,29,30, 33,38,39], three in villages [28,34,37], two in both rural and urban areas [24, 36] and for two studies, no description of the study site was available[25, 32] (Table 2 for more detailed description).

#### Interventions

Of the four Intervention Control Studies [25, 28, 30, 32], no interventions were applied in the control group of one study [28]. Ultra low volume (ULV) pyrethroid thermal fogging [25], *Bti* and Methoprene-S [30] and 4% Malathion fog [32] were applied in the control groups of the other three Intervention Control studies. Among the three Cluster Randomised Trials [29,33,39], two studies [29, 39] used temephos in combination with other interventions as a control group for a different intervention under study. Health education with source reduction was applied in the control group of the third cluster randomised trial[33].

Sample size was highly variable, with the number of containers or premises varying from 235 [24] to the coverage of entire islands[30, 31, 35].Follow up periods ranged from 6months [28] to 9 years [34] post intervention. Only one study [25] used a parameter for dengue transmission (DHF), measuring in communities the basic knowledge of dengue in relation to previous occurrence of DHF cases.

Temephos application was most commonly combined with health education and information, education and communication (IEC) activities [24,27,38,33, 34,36,37,38,39], and three studies [27,38, 39]included the use of mass media. Environmental management [24,27,29,30,33, 34,39] and malathion adulticide applications[26,32,35,37,38] were also used in combination with temephos. Four studies used biological control methods such as larvivorous fish in potable water[34] and in non-potable water[25], and *Bti*[27,30,34]. Two studies used mechanical interventions[28, 29]. Additional chemical insecticides, including methoprene-S, permethrin, cypermethrin and clorpyriphos, were used in five of the studies[25,30,31,36,39].

All combination intervention studies except for two[24, 25]used temephos at a dose of 1ppm. All studies used temephos sand granules except for two studies [24, 35],which used temephos as emulsifiable concentrate. Only in study [30] temephos had been provided routinely before the intervention. In study [38],temephos was supplied for a nominal fee to households. In study [36],temephos was partially substituted with methoprene and in study[34],temephos was replaced with *Bti* because of its unpleasant odour. In study[27], the focal treatment with temephos was interrupted due to a dengue outbreak and supplemented with ULV spraying.

Most studies [24,25,26,27,29,33,34,35,36,37,38,39]used different community members/groups either as a target group for education or for the delivery of the intervention. Study [39] formed a community working group exclusively for the implementation of the control activities in order to analyse the importance of community participation.

#### Community-effectiveness

Of 16 studies, 10[24,25,26,28,29,32, 34,35,38,39] reported a significant reduction post-intervention of immature *Aedes* stages as compared to baseline findings and control groups. Three studies [30,31,33] failed to reduce *Aedes* populations. Study [27]reported an initial drop in larval indices, however this was not sustained. Although larval breeding was reduced in study [36], the BI remained greater than 100 throughout the year. Study [37] reported only moderate effects of temephos on entomological indices.

For example, the effect of temephos on larvae in study [27] lasted 6 weeks, with infestations in treated containers detected after 6 weeks of treatment, whereas study [28]showed an effect of 6 months, with an observed increase of adult mosquito population 6–7 weeks post-treatment. In study [32], the effect on larvae lasted 8 weeks, study [35] showed a variation between 2–8 weeks, and for study [38] the effect waned after 3 months.

Four studies stratified their analyses according to the individual effect of each intervention, showing that temephos was the most effective intervention alone [32] or in combination with source reduction [24, 30] and ULV spraying [27].

While 10studies generally reported positive community-effectiveness parameters of the interventions [24,25,26,28,29,34,35,37,38,39], six studies [27,30,31,32,33,36]reported shortcomings related to attaining effectiveness.

From those reporting positive results, however study [28], while reporting successful outcomes in the short term, concluded that long term success depended on political commitment and community participation. Study[29] showed that factors such as high water turnover and temephos resistance in local vector populations contributed to decreased community-effectiveness. Another limiting factor was the limited knowledge of people about the interventions [25, 38].

In the group of studies reporting shortcomings these included problems related to surveillance and coverage[27],inadequate source reduction and larvicidal application [30], low acceptability of larvicides [36] and lack of resources and manpower [31].Other challenges included the limited residuality of temephos [27] and overflowing of water in containers resulting in consequent dilution of the active ingredient[33].

One important challenge raised regarding the use of temephos in combination interventions described in both groups was the false sense of security that can arise, leading the population to believe that temephos application alone is sufficient to prevent dengue which resulted in foregoing activities such as source reduction and other environmental management [24,33].

#### Acceptability

Acceptability of temephos was not assessed in all studies but was found to be low in three studies [34,36,37] due to unpleasant odour. For this reason, study [34] replaced temephos with *Bti* as an intervention, and in study[36],temephos was partially substituted with methoprene. Also, the application of temephos was found to be labour intensive in study[24].

## Discussion

### Limitations of the study

Studies on the efficacy of temephos were not assessed and only its community-effectiveness was analysed. However, temephos is used routinely in many parts of the world as a part of dengue vector control activities, and hence this systematic review focused on evidence for its community-effectiveness in the manners in which it has been applied routinely. Although we used a broad search strategy we could have missed potentially relevant studies. We also assume some publication bias towards studies demonstrating a positive effect.

The variability of the outcome measures encountered and the different larval and pupal indices used also limit the comparability of the studies, especially when relating outcomes to dengue transmission[40]. Moreover, very few studies monitored the changes in reported cases of dengue. Among the combined intervention studies, only four studies clearly stated which interventions were found to be effective, while the rest of the studies failed to disaggregate the data.

### General discussion

Overall, a diverse picture emerged with regards to the community-effectiveness of temephos reported in the 27 reviewed studies. Whilst the single intervention studies showed consistently that using temephos led to a reduction of entomological indices, this did not always hold true when applying temephos in combination with other interventions. For the latter group, three studies [30, 31, 33] clearly stated that temephos application along with other chemical vector control methods failed to reduce the larval and pupal indices, and while a further 10 studies reported a post-intervention reduction in immature mosquito stages, the results were either not sustainable over time or the coverage was not complete. The reasons for this can be manifold, and this has important implications for dengue control programmes and raises further questions: were the reasons operational, since when applying combined interventions the focus may perhaps shift from quality to quantity, or are there yet unknown interactions between the different interventions? Or was it simply because of limited resources? A further implication for dengue control programmes is the unknown epidemiological impact of temephos-based interventions, only one study linked basic knowledge of dengue to a reduction of DHF [25].

The effectiveness of temephos interventions depends on many factors related to the quality of delivery and maintenance of the intervention, including water turnover rate and type of water, as well as environmental factors such as organic debris, temperature and exposure to sunlight. This suggests that quality control and the suitability of application sites are key to the ultimate success of the intervention. However, the reviewed studies reporting the residual effect of temephos showed a duration between two and three months, confirming similar findings upon which operational guidelines have been based[9]. This recommended periodicity could have practical implications, however, as frequent re-treatment led to reluctance of temephos use in one of the reviewed studies[22]. Another study[19] suggested that a combined method of cyclic mass treatment at three month intervals with targeted treatment of new habitats between the mass treatments would offer optimal control.

The timing of the use of temephos is another factor to consider: dengue epidemics tend to occur in warm, humid and rainy seasons, favouring the growth of mosquito populations[41]. Hence, the timing of temephos applications can influence the efficacy of dengue vector control, as demonstrated by studies [14,18,23]which suggested that temephos application at the beginning of the rainy season was most likely to control the occurrence of an epidemic, while two studies [16,20]reported the successful control of *Aedes* species during both the dry and wet seasons.

The method of temephos application recommended by WHO is the use of calibrated plastic spoons to apply the appropriate doses to water-holding containers [9]. However, this review has shown that many different formulations and methods of temephos application can be successfully used, including zip-lock bags [13]which had longer residual effects and were cheap and easy to apply. The application of temephos using spoons [13,18]was reported to create an unpleasant odour, taste and turbidity-a disadvantage not seen with temephos zeolite formulations [15]. Using corncorb grit granules with a blower [21]was cheaper, required less manpower and was effective due to its dispersion. In the *Ae*. *Aegypti* elimination programme carried out on an island, many temephos delivery systems were successfully applied, such as emulsion paint on walls, perifocal application of untreated areas, larviciding of water containers[22]. In one of the reviewed studies [21], temephos was also found to be effective when it was applied at half the recommended dosage.

Coverage of potential breeding sites is a recurrent issue: inaccessibility of breeding sites for temephos treatment can be important in cases such as leaf axils, brick or rock holes, miscellaneous containers such as cans, bottles, tiers, flower vases etc. Another aspect of coverage is the question of distance between the treated household and untreated areas. In a study related to *Ae*. *aegypti* dispersal, Reiter *et al*. [42] showed that maintaining a treated barrier zone of 50–100 meters around the house of a dengue case is unlikely to be effective, as *Ae*. *aegypti* can fly much further to oviposit. This was evident in one of the studies [16]where there-infestation of a treated area occurred despite the maintenance of a barrier zone of 87 houses.

Operational aspects, such as cost, supplies, time and labour efforts are further issues limiting the potential effectiveness of temephos application [19,29],as is low community acceptability(such as reluctance to use temephos in drinking water)[15, 24,34, 36, 37]. These factors are very important considering that for any community based programme to be successful, it needs to be widely accepted by the people with whom the intervention is being carried out[43],and that programmes need to reflect the "felt needs" of the people in order to maintain their interest, motivation and long-term engagement[44].

No conclusive evidence was attained regarding which intervention was the most effective, nor was it possible to come to a conclusion regarding which combinations of interventions formed the best package in terms of effectiveness, feasibility, cost and sustainability. For the group of studies failing to show effectiveness of temephos combined with other interventions, the failure was attributed to false complacency arising from the perception that temephos was sufficient to control the vector, neglect of source reduction activities [22] and low acceptability of temephos application in potable water[36]. This highlights the importance of community participation, as has been proposed with "Communication-for-Behavioural-Impact" (COMBI) efforts[45].The findings of this study on community participation were similar to those of a systematic review conducted by Heintze *et al*.[46],which suggested that community-based control strategies implemented together with other interventions were able to reduce classical *Aedes* larval indices, but they were unable to disentangle the effect of different interventions and community participation.

The role of entomological surveillance is another important factor: WHO recommends the implementation of an integrated dengue surveillance and outbreak preparedness system[47]. The importance of implementation and maintenance of a surveillance programme was highlighted in study [22], which reported the importation of viable *Ae*.*aegypti* eggs through a crate of household articles and through trade delivered via the ports.

In conclusion, this review presents more questions than answers regarding the community-effectiveness of temephos for dengue vector control. While there is little doubt concerning the effectiveness of temephos in controlling *Aedes* breeding sites, the same level of effectiveness was not clear from the studies using temephos combined with other interventions. No conclusive evidence has been shown regarding the impact of temephos interventions on dengue transmission.

This review highlights that although temephos is one of the most widely used interventions against dengue vectors worldwide, its effectiveness can vary greatly. The lack of data relating reductions in entomological indices to reductions in dengue transmission remains a significant knowledge gap in the area of dengue epidemiology and vector control efficacy. Integrated surveillance activities may need to be implemented along with vector control interventions, in order to address this knowledge gap.

## Supporting Information

S1 Checklist PRISMA. (DOC)

## References

[1] WHO (2009) Dengue: guidelines for diagnosis, treatment, prevention and control WHO 2009, Geneva, Switzerland ISBN 978 92 4 154787 1. http://whqlibdoc.who.int/publications/2009/9789241547871_eng.pdf (accessed 07.03.15)

[2] WHO (1982) Manual on environmental management for mosquito control WHO, Geneva whqlibdoc.who.int/publications/1982/9241700661_eng.pdf (accessed 07.03.15)

[3] WHO (2004) Global strategic framework for integrated vector management WHO, Geneva, Switzerland http://whqlibdoc.who.int/hq/2004/WHO_CDS_CPE_PVC_2004_10.pdf (accessed 07.03.15)

[4] Esu E, Lenhart A, Smith L and Horstick O (2010). Effectiveness of peridomestic space spraying with insecticide on dengue transmission; systematic review, TMIH, Volume 15 No 5 PP 619–631 5 2010 10.1111/j.1365-3156.2010.02489.x 20214764

[5] Boyce R, Lenhart A, Kroeger A, Velayudhan R, Horstick O Bacillus thuringiensis israelensis (Bti) for the control of dengue vectors: A systematic review TMIH Volume 18, Issue 5, pp 564–577, 5 2013 10.1111/tmi.12087 23527785

[6] Sztankay-Gulyás M (1972) Mosquito control with integrated method. Wiadomosci Parazytologiczne, 1972, 18, 629–33. 4122220

[7] WHO (2009) Temephos in drinking water: Use for vector control in drinking- water sources and containers . Background document for development of WHO Guidelines for drinking-water quality. WHO, Geneva http://www.who.int/water_sanitation_health/dwq/chemicals/temephos.pdf (accessed 07.03.15)

[8] WHO (2008) WHO Specifications and evaluations for public health pesticides: Temephos WHO, Geneva http://www.who.int/whopes/quality/Temephos_eval_only_oct_2008.pdf (accessed 07.03.15)

[9] SEARO WHO (2011) Prevention and Control of Dengue and Dengue Haemorrhagic Fever: Comprehensive Guidelines. SEARO, New Delhi http://apps.searo.who.int/pds_docs/B4751.pdf?ua=1 (accessed 07.03.15)

[10] Pinheiro VCS, Tadei P (2002) Evaluation Of The Residual Effect Of Temephos On Aedes Aegypti (Diptera, Culicidae) Larvae In Artificial Containers In Manaus, Amazonas State, Brazil. Cad. Saúde Pública, Rio De Janeiro, 01/2002, 18 (6), 1529–1536. 10.1590/s0102-311x2002000600005 12488878

[11] Moher D, Liberati A, Tetzlaff J, Altman DG, The Prisma Group (2009) Preferred Reporting Items for Systematic Reviews and Meta-Analyses: The PRISMA Statement. PLoS Med 6(7). PLoS Med 6(6): e1000097. 10.1371/journal.pmed1000097 PMC2707599 19621072

[12] Shea BJ, Grimshaw JM, Wells G A, Boers M, Anderson N et al (2007) Development of AMSTAR: a measurement tool to assess the methodological quality of systematic reviews. BMC Medical Research Methodology,7,10 http://www.biomedcentral.com/1471-2288/7/10 (accessed 07.03.15) 17302989 10.1186/1471-2288-7-10 PMC1810543

[13] Garelli FM, Epinosa MO, Weinberg D, Trinelli MA, Gürtler RE (2011) Water Use Practices Limit the Effectiveness of a Temephos-Based Aedes aegypti Larval control program in Northern Argentina. PLoS Neglected Tropical Diseases, 2011, 5(3):e991 http://journals.plos.org/plosntds/article?id=10.1371/journal.pntd.0000991 (accessed 07.03.15) 21445334 10.1371/journal.pntd.0000991 PMC3062537

[14] Vezzani D, Velázquez SM, Schweigmann N (2004) Control of Aedes aegypti with temephos in a Buenos Aires cemetery, Argentina. Revista de Saúde Pública, 2004, 38(5),738–740. 15499449 10.1590/s0034-89102004000500020

[15] Thavara U, Apiwat T, Kong-Ngamsuk W, Mulla MS (2004). Efficacy and longevity of a new formulation of Temephos larvicide tested in village-scale trials against larval Aedes Aegypti in water-storage containers. Journal of the American Mosquito Control Association, 2004, 20(2), 176–182. 15264628

[16] Geevarghese G, Dhanda V, Ranga Rao PN, Deobhankar RB (1977). Field trials for the control of Aedes aegypti with abate in Poona city and suburbs. The Indian Journal of Medical Research, 1977, 65(4), 466–473. 71265

[17] Garza-Robledo AA, Martinez JF, Violeta A, Rodríguez-Castro, Quiroz-Martinez H (2011) Effectiveness of Spinosad and Temephos for the control of mosquito larvae at a tire dump In Allende, Nuevo Leon,Mexico. Journal of the American Mosquito Control Association, 2011, 27(4), 404–407. 22329273 10.2987/11-6133.1

[18] Bang YH, Pant CP (1972a) A field trial of Abate larvicide for the control of Aedes aegypti in Bangkok, Thailand. Bulletin of the World Health Organization, 46(3), 416–425. 4537857 PMC2480745

[19] Bang YH, Pant CP (1972b) Pilot studies of Abate as a larvicide for control of Aedes aegypti in Bangkok, Thailand. Southeast Asian J Trop Med Public Health, 1972, 3(1), 106–115. 5028856

[20] Marina CF, Bond JG, Casas M, Muñoz J, Orozco A, et al (2011) Spinosad as an effective larvicide for control of Aedes albopictus and Aedes aegypti, vectors of dengue in southern Mexico. Pest Management Science, 2011, 67, 114–121. 10.1002/ps.2043 21162151

[21] Morris CD, Dame DA, Robinson WJ (1996) Control of Aedes Albopictus in waste tire piles with reduced rates of Temephos-treated granules. Journal of the American Mosquito Control Association, 1996, 12(3), 472–476. 8887227

[22] Nathan MB, Giglioli MEC (1982) Eradication of Aedes Aegypti on Cayman Brac and Little Cayman, West Inidies, with Abate (Temephos) in 1970–1971. Bulletin of the Pan American Health Organization, 1982, 16(1), 28–38. 6176286

[23] Chadee DD (2009) Impact of pre-seasonal focal treatment on population densities of the mosquito Aedes aegypti in Trinidad, West Indies: A preliminary study. Acta Tropica, 2009, 109, 236–240. 10.1016/j.actatropica.2008.12.001 19114025

[24] Shriram AN, Sugunan AP, Manimunda SP, Vijayachari P (2009) Community-centred approach for the control of Aedes spp. in a peri-urban zone in the Andaman and Nicobar Islands using temephos. The National Medical Journal of India, 2009, 22(3), 116–120. 19764685

[25] Chaikoolvatana A, Chanruang S, Pothaled P (2008) A comparison of dengue hemorrhagic fever control interventions in northeastern Thailand. The Southeast Asian Journal of Tropical Medicine and Public Health, 2008, 39(4), 617–624. 19058598

[26] Laws ER, Sedlak VA, Miles JW, Joseph CR, Lacomba JR et al (1968) Field study of the safety of abate for treating potable water and observations on the effectiveness of a control programme involving both Abate and Malathion. Bulletin of the World Health Organization, 1968, 38(3), 439–445. 5302335 PMC2554476

[27] Gürtler RE, Garelli FM, Coto HD (2009) Effects of a five-year citywide intervention program to control Aedes aegypti and prevent dengue outbreaks in Northern Argentina. PLoS Neglected Tropical Diseases, 2009, 3(4):e427 http://journals.plos.org/plosntds/article?id=10.1371/journal.pntd.0000427 (accessed 07.03.15) 10.1371/journal.pntd.0000427 19399168 PMC2669131

[28] Lardeux F, Sechan Y, Loncke S, Deparis X, Cheffort J et al (2002) Integrated control of peridomestic larval habitats of Aedes and Culex Mosquitoes (Diptera: Culicidae) in Atoll villages of French Polynesia. Journal of Medical Entomology, 2002, 39(3), 493–498. 12061446 10.1603/0022-2585-39.3.493

[29] Rizzo N, Gramajo R, Escobar M, Arana B, Kroeger A et al (2012) Dengue vector management using insecticide treated materials and targeted interventions on productive breeding-sites in Guatemala BMC Public Health, 2012, 12:931 http://www.biomedcentral.com/1471-2458/12/931 (accessed 07.03.01) 10.1186/1471-2458-12-931 23110515 PMC3533994

[30] Favier C, Degallier N, Vilarinhos P, Carvalho M, Yoshizawa M et al (2006) Effects of climate and different management strategies on Aedes aegypti breeding sites: a longitudinal survey in Brası´lia (DF, Brazil). Tropical Medicine and International Health, 2006, 11(7), 1104–1118. 16827711 10.1111/j.1365-3156.2006.01653.x

[31] Wheeler AS, Petrie WD, Malone D, Allen F (2009) Introduction, control, and spread of Aedes albopictus on Grand Cayman Island, 1997–2001. Journal of the American Mosquito Control Association, 2009, 25(3), 251–259. 19852213 10.2987/08-5794.1

[32] Bang YH, Gratz N, Pant CP (1972b) Suppression of a field population of Aedes aegypti by malathion thermal fogs and Abate larvicide. Bulletin of the World Health Organisation, 1972, 46(4), 554–558 PMC2480780 4538200

[33] Camargo Donalisio MR, Ferreira Leite O, Caporale Mayo R, Chinelatto Pinheiro Alves MJ, de Souza A et al (2002) Use of Temephos for control of field population of Aedes aegypti in Americana São Paulo, Brazil. Dengue Bulletin, 2002, 26, 173–177. http://apps.searo.who.int/pds_docs/B0222.pdf (accessed 07.03.15)

[34] Wang CH, Chang NT, Wu HH, Ho CM (2000) Integrated control of the dengue vector Aedes aegypti in Liu-Chiu village, Ping-Tung County, Taiwan. Journal of the American Mosquito Control Association, 2000,16(2), 93–99. 10901632

[35] Gould DJ, Scanlon JE, Sullivan MF, Winter PE (1971) Dengue control on an island in the Gulf of Thailand. The American Journal of Tropical Medicine and Hygiene, 1971, 20(5), 705–714. 5093668 10.4269/ajtmh.1971.20.705

[36] Phanthumachinda B (1984) Pilot studies on community participation: Aedes Aegypti control at Phanus Nikhom District, Chonburi Province,Thailand: Frist Year Report. Dengue Bulletin, 1984, 10, 35–41.

[37] Eamchan P, Foy HM, Chareonsook OA (1989) Epidemiology and control of dengue virus infections in Thai villages in 1987. American Journal of Tropical Medicine and Hygiene, 1989, 41(1), 95–101. 2764233

[38] Swaddiwudhipong W, Chaovakiratipong C, Nguntra P, Koonchote S, Khumklam P et al (1992) Effect of health education on community participation in control of Dengue Hemorrhagic Fever in an urban area of Thailand. Southeast Asian J Trop Med Public Health, 1992, 23(2), 200–206. 1439971

[39] Vanlerberghe V, Toledo ME, Rodríguez M, Gomez D, Baly A et al (2009) Community involvement in dengue vector control: cluster randomised trial. BMJ, 2009, 338: b1959 http://www.bmj.com/content/338/bmj.b1959 (accessed 07.03.15) 10.1136/bmj.b1959 19509031 PMC2694260

[40] Sivagnaname N, Gunasekaran K (2012) Need for an efficient adult trap for the surveillance of dengue vectors. The Indian Journal of Medical Research, 2012, 136(5): 739–749. 23287120 PMC3573594

[41] Gibbons RV and Vaughn DW (2012) Dengue an escalating problem. BMJ 2002 Jun 29; 324(7353):1563–6 10.1136/bmj.324.7353.1563 PMC1123504 12089096

[42] Reiter P, Manuel A, Robert A, Anderson A, Clark G (1995) Short Report: Dispersal of Aedes aegypti in an urban area after blood feeding as demonstrated by Rubidium—Marked Eggs. American Journal of Tropical Medicine and Hygiene 1995, 52 (2), 177–179. 7872449 10.4269/ajtmh.1995.52.177

[43] Rifkin S (1986) Lessons from community participation in health programmes. Health policy and planning, 1986, 1 (3), 240–249.

[44] Onyenemezu E, Olumati ES (2013) The Imperativeness Of Felt-Needs In Community Development. Journal Of Education And Practice, 2013, Vol 4, No 2, 156–159 http://www.iiste.org/Journals/index.php/JEP/article/view/4083/4116 (accessed 07.03.15)

[45] Lloyd L, Parks W (2004 ) Planning social mobilization and communication for dengue fever prevention and control: A Step- By-Step Guide WHO, Geneva http://www.who.int/immunization/hpv/communicate/planning_social_mobilization_and_communication_for_dengue_fever_prevention_and_control_who_cds_wmc_2004.pdf

[46] Heintze C, Velasco Garrido M, Kroeger A (2007) What do community-based dengue control programmes achieve? A systematic review of published evaluations. Transactions of the Royal Society of Tropical Medicine and Hygiene, 2006, 101 (4), 317–325 17084427 10.1016/j.trstmh.2006.08.007

[47] WHO (2012) Global Strategy for dengue prevention and control 2012–2020, http://apps.who.int/iris/bitstream/10665/75303/1/9789241504034_eng.pdf (Accessed 07.03.15)

